# Chinese physicians’ perceptions of palliative care integration for advanced cancer patients: a qualitative analysis at a tertiary hospital in Changsha, China

**DOI:** 10.1186/s12910-022-00750-7

**Published:** 2022-03-04

**Authors:** Jessica Hahne, Xiaomin Wang, Rui Liu, Yuqiong Zhong, Xin Chen, Xing Liu, Kaveh Khoshnood, Xin Li

**Affiliations:** 1 Center of Clinical Pharmacology, The Third Xiangya Hospital of Central South University, Changsha, 410013 People’s Republic of China; 2 Center for Medical Ethics, Central South University, Changsha, 410013 People’s Republic of China; 3 Department of Hematology, The Third Xiangya Hospital of Central South University, Changsha, 410013 People’s Republic of China; 4 School of Public Administration, Central South University, Changsha, 410075 People’s Republic of China; 5 Xiangya Hospital of Central South University, 87 Xiangya Road, Changsha, 410008 Hunan People’s Republic of China; 6 Yale School of Public Health, New Haven, CT USA

**Keywords:** China, Cancer, Communication, Ethics, Decision making, Palliative care

## Abstract

**Background:**

Little previous research has been conducted outside of major cities in China to examine how physicians currently perceive palliative care, and to identify specific goals for training as palliative care access expands. This study explored physicians’ perceptions of palliative care integration for advanced cancer patients in Changsha, China.

**Methods:**

We conducted semi-structured qualitative interviews with physicians (n = 24) specializing in hematology or oncology at a tertiary hospital.

**Results:**

Most physicians viewed palliative care as equivalent to end-of-life care, while a minority considered it possible to integrate palliative care with active treatment. Almost all physicians maintained separate conversations about palliative care with family members and patients, communicating more directly with family members than with patients about prognosis and goals of care. Physicians described experiencing ethical tension between the desire of family members to protect the patient from knowing they have advanced cancer, and the patient’s “right to decide” about palliative treatment. Physicians varied overall regarding perceptions of the role they should have in discussions about goals of care.

**Conclusions:**

As palliative care access expands in China, medical training should encourage earlier integration of palliative care for advanced cancer, address ethical issues faced by physicians communicating about palliative care, and establish guidance on the role of the physician in discussions about goals of care.

## Background

Cancer is a leading cause of death in China, a country that represents nearly one-fifth of the world’s population [1, 2]. Palliative care aims to support quality of life by addressing the range of physiological, psychological, social, and spiritual issues experienced by patients with serious illnesses, including but not limited to cancer [3, 4]. For advanced cancer patients, research increasingly shows integrating palliative care into treatment as early as initial diagnosis can increase quality of life, decrease risk of depression and anxiety, improve family satisfaction with care, and even prolong survival [5–10]. However, a report by *The Economist* in 2015 characterized China as “facing difficulties from slow adoption of palliative care and a rapidly aging population,” and ranked China 71st out of 80 countries on quality of palliative care [11, 12].

Research suggests that slow adoption of palliative care in China is an issue of both access and training. Many places outside of major cities lack dedicated palliative care facilities, and few rural areas have sufficient access to pain medication [13, 14]. Government initiatives in 2011 and 2017 established about 913 pilot hospital units for cancer pain management, and 71 pilot areas for hospice services throughout China [1, 13]. However, shortcomings in palliative care training may be causing barriers to persist, even as resources start to expand. According to recent surveys, less than 15% of medical interns in China feel sufficiently trained in basic pain and symptom management [2], and 69% of oncologists have had no palliative care training [15].

Palliative care training programs in various countries emphasize competencies not only in pain management, but also in communication and ethics [16, 17]. Physicians providing palliative care must be able to communicate effectively about changes in the patient’s condition and treatment options, in addition to managing pain and symptoms [18]. Effective palliative care also requires physicians to be competent in managing ethical dilemmas and providing care in a manner that is sensitive to the values of patients and families [19].

Research suggests physicians in China frequently face dilemmas in communication and ethics when caring for patients with serious illnesses—particularly cancer, which has a strong connotation of being life-threatening. Traditional Chinese culture treats discussion of death, and therefore life-threatening illness, as taboo [1]. In China’s family-centered culture, physicians routinely inform families of cancer diagnoses first, allowing families to decide whether or when to disclose information to patients. Families often withhold information from patients, desiring to protect patients from despair [20, 21]. But medical law in China has been changing in recent years to emphasize individual informed consent [21], and individuals increasingly report that they would want to know if they had cancer [22]. In this changing context, doctors report experiencing ethical tension between honoring the family’s preference for protective nondisclosure and honoring the patient’s “right to know” about their condition [21].

Little research has examined how cancer communication dynamics in China may affect palliative care integration. International oncology guidelines are only beginning to standardize recommendations for the timing of integrating palliative care into cancer treatment—with a 2021 study showing that almost half of National Comprehensive Cancer Network guidelines in the US lacked any mention of palliative care [23]. Given the additional absence of standard guidelines in most Chinese hospitals [24], more research is needed to understand how physicians currently approach discussions about palliative care with advanced cancer patients and families, in order to inform effective palliative care training. While previous surveys have examined Chinese physicians’ palliative care knowledge and skills, this study provides the first in-depth, qualitative analysis of Chinese physicians’ perceptions of palliative care integration for advanced cancer patients.

## Methods

### Study design, location, and participants

We conducted in-depth, semi-structured interviews with physicians at a tertiary hospital in Changsha, Hunan Province, south-central China. Tertiary hospitals in China are at the top of a three-level classification system ranking hospitals by ability to provide medical care, education, and research. Tertiary hospitals frequently serve as regional hubs for comprehensive, specialized medical care [25]. The China Business Network Research Institute publishes a city classification system that ranks Changsha as a “new first-tier city,” one classification below the four “first-tier cities” of Beijing, Shanghai, Guangzhou, and Shenzhen in terms of development [26]. We chose a tertiary hospital in Changsha as the study location, because less is known about palliative care in cities below the first tier, where specialized palliative care units are less common [14].

Physicians were recruited according to the following inclusion criteria: aged 18 or older, from the department of hematology or oncology, and with at least three years’ experience caring for cancer patients. We recruited participants until reaching data saturation, at which point no new themes were emerging from the data [27]. A total of 24 out of 25 physicians we contacted agreed to participate (96%), with one physician declining due to lack of time. A majority of participants were female residents, attending physicians, or professors. Participants had varying levels of education and work experience. A complete description of participant characteristics can be viewed in Table 1.

**Table 1:** Characteristics of 24 study participants

Characteristics	Value (%)
*Department*	
Hematology	14 (58.3)
Oncology	10 (42.7)
*Gender*	
Male	3 (12.5)
Female	21 (87.5)
*Education level*	
Bachelor’s degree	1 (4.2)
Master’s degree	8 (33.3)
Doctoral degree	15 (62.5)
*Job status*	
Resident doctor	10 (41.6)
Attending doctor	7 (29.2)
Professor	7 (29.2)
*Work experience (years)*	
3–5	9 (37.5)
5–10	5 (20.8)
> 10	10 (41.7)

### Data collection

Interviews for this study were conducted remotely by video call, via the mobile app WeChat, between April 2020 and September 2020. All authors met together to design an interview guide investigating cancer physicians’ perceptions of palliative care integration. All questions from the finalized interview guide are listed in Table 2. Interviews were conducted in Mandarin Chinese by authors X. Wang and R. Liu. The research team discussed possible probes and follow-up questions before beginning interviews, and interviewers used them when necessary to draw out more information relevant to the main research question. The second interviewer observed the first interviewer before beginning interviews, in order to standardize approaches and minimize bias. Oral informed consent was obtained from all participants. Interview recordings were stored on a secure, password-protected computer. Interviews ranged from 20 to 50 min long.

**Table 2:** Semi-structured interview guide

1. Can you describe your views on palliative care for patients with advanced cancer?
2. Can you describe your opinion on different treatment options for advanced cancer? Do you prefer a more active treatment plan, or a more palliative treatment plan?
3. Would you recommend that patients with advanced cancer receive palliative care?
4. When you make a decision to recommend palliative care for patients with advanced cancer, what are the main considerations?
5. At what stage do you think patients with advanced cancer should receive palliative care? Would you recommend palliative care at the patient's first visit?
6. How do you inform advanced cancer patients who need palliative care?
7. What factors do you think will affect the decision of advanced cancer patients and their families, as to whether to choose palliative care?
8. Can you describe how you would deal with a situation in which you would recommend that patients receive palliative care, but the patient or family members still request only active treatment?
9. Can you describe your views on the use of pain medications, and pain-related issues for palliative care patients?
10. What do you think are ethical issues in this area? Can you describe any ethical issues you have encountered related to palliative care?
11. Can you describe how you solve issues related to palliative care and ethics? What guides you to solve this type of problem?
12. What palliative care skills and ethical problem-solving skills do you think oncologists need to have?
13. Have you received training on palliative care and ethical issues?
14. Do you think it is necessary to promote and improve the palliative care of patients with advanced cancer in China?
15. What do you think is the current status of the development of palliative care for advanced cancer patients in China? Are there any obstacles or challenges?
16. Do you have anything to add regarding palliative care and ethical issues?

### Data analysis

Recorded interviews were transcribed verbatim in Mandarin. Interview transcripts were independently coded by authors R. Liu and Y. Zhong. Data was analyzed by conventional content analysis, which avoids using pre-conceived categories to generate codes. This modality is considered appropriate when current knowledge on the phenomenon being researched is limited [28]. The authors derived initial codes from major concepts relevant to the research question that emerged from interview data. Initial codes were discussed among all authors, who reconciled any differences in team discussions centered on the research question and relevant sub-questions. A finalized codebook including 17 codes and 62 sub-codes was used to code all interviews, using NVivo 11 software. The authors used constant comparison to check for consistent application of codes across transcripts.

All coded segments of interview data were translated into English by authors L. Rui and Y. Zhong, native Mandarin speakers, and double-checked for accuracy by author J. Hahne, a native English speaker. Concepts that emerged through coding were organized through team discussion into three main categories: physicians’ conceptualizations of palliative care, approaches to palliative care communication, and self-perceived roles in discussions about goals of care.

## Results

### Conceptualizations of palliative care: an “end of the line” alternative, versus an “important part of comprehensive treatment”

While doctors generally agreed palliative care could help relieve suffering for advanced cancer patients, two contrasting frameworks for its purpose and timing emerged across interviews. Most doctors conceptualized palliative care as a last resort, equating it with end-of-life care and considering it only when they could not provide active treatment. Only a few doctors believed palliative care could be complementary and integrated with active treatment.

Doctors who saw palliative care as a last resort described it using phrases such as *“there’s no other way”* (Participant 12 [P12]), *“really at the end of the line,”* (P18), and *“for older patients or those with poor basic conditions”* (P20). They said they were *“inclined toward active treatment”* (P16), and viewed palliative care as conflicting with goals of active treatment: *“If the patient can still go to chemotherapy, then palliative care is given too early, as if he will not necessarily benefit (from active treatment)”* (P13). They described turning to palliative care for two reasons: if active treatment was too expensive, or if the patient had an especially poor prognosis (P13). They also did not believe palliative care had any potential to prolong survival:

*“[The palliative care patient] is a patient with advanced cancer whose survival cannot be prolonged by our usual anti-tumor methods. In this case, we may try to relieve the pain with some symptomatic support treatment instead of aggressive treatment […] because there is no way to prolong his life in palliative care.”* (P17)

By contrast, a minority of doctors viewed palliative care as an *“important part of comprehensive treatment”* (P05), and considered the goals of palliative and active treatment to be *“mutually transformative”* (P01). Unlike doctors in the first group, these doctors acknowledged that palliative care could help lengthen survival in some cases: *“Palliative care is to alleviate the suffering of the patient. If it can prolong his life, even better. If it can't be prolonged, it's still a relief to the patient”* (P03).

One doctor in the second group recommended initiating palliative care for the same main reasons as doctors in the first group: when active treatment was too expensive, or the patient’s prognosis especially poor (P21). However, she conceptualized palliative care as integrated with active treatment: *“Palliative care is giving patients palliative treatment in addition to cancer treatment—to not only control physical pain and treatment side effects, but also support psychological problems and improve quality of life”* (P21). Other doctors in the second group placed less emphasis on prognosis, stating palliative care could begin *“from the moment of diagnosis”* (P02) or *“from an early stage”* (P16).

Despite their contrasting views, at least one doctor in each group desired training on the ideal timing of palliative care integration for advanced cancer patients (P13, P21). Overall, only 5 of 24 doctors stated that they had received any training in palliative care.

### Approaches to communication: How concealing advanced cancer diagnoses leads to “two sets of dialogue” with patients and families about palliative care

Although conceptualizations of palliative care differed among doctors, approaches to communication were remarkably uniform. From the beginning, doctors followed the preference of most families to conceal the patient’s diagnosis, at least in pretense: *“If the family asks me to conceal [the diagnosis] from the patient, I will withhold it. But because by this time, in fact, most patients are symptomatic, I think it would be very difficult in practice to completely conceal it”* (P05). Preserving this pretense over the course of treatment required doctors to engage in *“two sets of dialogue”* about treatment options—one with the patient, and one with the family:

*“We actually have two sets of dialogue. When you talk to a patient, there are some things that you might not mention. […] I will talk less about the bad, and I will certainly try to give the patient as much hope as possible. […] But when talking to the family, due to the way the medical environment is, you can't give the family too much hope. Because if you let the family hold expectations too high, if you do not meet them in the end, it will cause medical disputes. So we talk more about bad things with the family.”* (P03)

When it came time to discuss palliative care, these *“two sets of dialogue”* emerged across interviews as a pattern of opposites. Doctors shared recommendations about palliative care *“first”* with families (P01, P05, P08, P13, P16), and more *“slowly”* with patients (P21, P24). They talked with patients *“mildly”* (P04), *“optimistically”* (P05), and *“euphemistically”* (P11); but with families in a way that was *“straightforward and direct,”* (P11), and *“realistic”* (P05, P13).

In addition to wanting to protect patients from despair (P04, P05, P09, P11, P13, P21), doctors worried if they did not frame information optimistically enough for patients, families who wanted to protect patients would raise disputes (P07, P13). They also worried that if they did not frame information pessimistically enough for families, families could be shocked if the patient declined, and accuse the doctor of malpractice (P03, P10).

At early stages of communication, some doctors described feeling ethical tension about compromising the patient’s *“right to know”* about their condition (P09, P21). As separate dialogues continued, they expressed concern about the patient’s *“right to decide”* between treatments, including palliative care (P14, P21). One doctor described how compromising the right to know evolved into compromising the right to decide: *“We have this problem in China: It seems that the patient's family, not the patient, decides his treatment, life, and death. The patient is not fully informed, and then perhaps he himself has little discretion in this matter”* (P07).

Several doctors desired training on ethics of palliative care communication (P01, P15, P18, P19, P21), both for patients’ benefit and to protect themselves from disputes: *“Particularly when opinions differ between doctors and families, it may be important for us to conduct ethical training on how to make treatment decisions to ensure the interests of patients without causing too much trouble to ourselves”* (P08).

### Self-perceived roles in discussions about goals of care: objective advisors, versus experts with influence

As doctors held separate conversations with patients and families about treatment options, they tended to view themselves according to one of two roles. While some doctors strove to be objective advisors, others saw themselves as experts with influence on discussions about goals of care.

Doctors who strove to maintain an objective role presented *“advice”* (P11, P23) or *“pros and cons”* (P16) about active and palliative treatment options to families, and sometimes to patients. They then drew boundaries for their involvement, stating: *“I don't have any subjective intentions to guide him (the patient),”* (P16) or, *“It is impossible for us to influence the patient's thoughts and ideas”* (P11). After initiating the conversation, they withdrew and left deliberations about goals of care to the family (P17). They also stayed out of conflicts that arose during families’ deliberations: *“They made their own arrangements and unified themselves. They didn't agree at first. Finally, the family came up with a consensus, including the patient and her immediate family”* (P23).

By contrast, a second group of doctors expressed awareness of their influence on patients’ and families’ decisions about treatment options, because of their role as experts. In some cases, their influence was implicit: “*The tone of the conversation can influence them a little bit. If curative treatment won’t have much significance, sometimes we talk more seriously”* (P13). In other cases, their influence was more intentional: *“If the doctor feels the patient needs to be treated [actively], the doctor might push the patient and the family over to that side”* (P04). When conflicts arose within families during discussions about goals of care, these doctors felt responsible to negotiate a consensus (P18).

Interestingly, some doctors considered it ideal to remain objective but felt that in practice, they could not avoid influencing patients’ and families’ decisions about treatment options. One physician stated at first that her goal was to *“let them (the family) make their own choices and not participate in their decisions,”* but later admitted, *“the doctor's own attitude toward the condition may directly influence the attitude of patients and their families”* (P07). Similarly, another doctor stated in one instance, *“I will speak up, but will not be involved in the decision,”* but noted later, *“Every patient has a different understanding of the concept of palliative care. […] If you tell him in a different way, it will influence his understanding”* (P05).

Doctors across both groups believed that training in psychology could help them play a more effective and empathetic role in discussions about goals of care (P01, P02, P09, P13, P23). Several also thought educating the general public about palliative care and death could foster more openness in palliative care communication and discussions about goals of care (P02, P10, P15, P24).

## Discussion

This study sheds light on previously under-researched aspects of palliative care in China, as both the first interview study to examine physicians’ perceptions of palliative care integration, and one of the few studies on palliative care conducted in China outside of first-tier cities. Findings revealed that doctors held contrasting views of palliative care as either equivalent to end-of-life care, or as an integrated part of comprehensive treatment for advanced cancer. While previous studies have shown concealing diagnoses from cancer patients is common practice in China, a novel finding in our study was the subsequent continuation of two separate dialogues about palliative care with families and with patients. A third novel finding was the contrast observed between doctors who viewed themselves as objective advisors in discussions about goals of care, versus as experts with influence—suggesting a range of possible views among doctors on the role they should play in discussions about goals of care. We raise several suggestions below on palliative care education and training, as well as directions for future research.

Doctors in this study who equated palliative care with end-of-life care echoed an older definition of palliative care from the WHO in 1990 that emphasized its use for “patients whose disease is not responsive to curative treatment” [29]. In 2002, the WHO updated its definition: “Palliative care is applicable early in the course of illness, in conjunction with other therapies that are intended to prolong life” [30]. Only a minority of doctors in our study understood palliative care by the newer definition. Oncology research in various countries suggests physicians’ understanding of palliative care as equivalent to end-of-life care can be a barrier to early use of palliative care for advanced cancer patients [9, 31, 32]. Because many patients in China already have advanced cancer at the time of diagnosis, there is particular need for palliative care early in treatment to aid quality of life [2]. Emphasizing the potential survival benefit of early palliative care may help counter the misconception of palliative care as end-of-life care. A widely cited 2014 randomized controlled trial among U.S. advanced cancer patients showed the median survival of patients receiving palliative care immediately after diagnosis was more than six months longer than patients who began palliative care three months after diagnosis [33]. In our study, only the few doctors who viewed palliative care by the newer definition believed palliative care could prolong survival.

Despite the clear benefits of early palliative care for quality of life and likely benefits for length of survival, palliative care referrals worldwide, including in first-tier cities in China, still generally occur late in the course of advanced cancer [24, 34–36]. Increasingly, leading oncology organizations are encouraging earlier integration of palliative care into advanced cancer treatment [37, 38]. More recently, the Chinese Society for Clinical Oncology also began to recommend early integration [39]. But the finding in our study that few doctors saw potential to integrate palliative care with active treatment suggests a gap persists between emerging guidelines and current training in China. As palliative care education and training expands in China, it is important for curricula not only to clearly distinguish between palliative and end-of-life care, but also to emphasize benefits of early palliative care for advanced cancer patients.

Our study also highlighted how the initial choice to conceal a cancer diagnosis from the patient at the family’s request leads physicians to face ethical concerns related to both patient autonomy, and beneficence. Concerns about patient autonomy arose as separate, ongoing conversations with families and patients developed around palliative care integration. Doctors moved from being concerned about compromising the patient’s “right to know” about their condition to compromising the patient’s “right to decide” between treatment options—essentially the defining aspects of patient autonomy in informed consent. While doctors in previous research in China have articulated concerns about the patient’s “right to know” about a cancer diagnosis and prognosis [21], the emergence of concerns later in treatment about the patient’s “right to decide” was a novel finding in our study. Regarding patients’ views on the “right to know,” as many as 98% of surveyed cancer patients in China believe patients should be informed of a cancer diagnosis [40], and as many as 91.9% believe patients should be informed of a terminal diagnosis [41]. Regarding the “right to decide” between treatment options, more research is needed to determine how directly cancer patients in China desire to engage in decisions [42]. Furthermore, culturally suitable communication models are needed to help physicians discern differences in individual patients’ preferences for disclosure and decision-making. The Japanese SHARE model for breaking bad news may be helpful to adapt in China, as it includes assessing how much each patient wants to know [43]. And as a model for decision-making, one research team has suggested family-centered care—a model common in pediatric medicine that views the whole family as the recipient of care—for Chinese oncology, as an alternative to the shared decision-making model popular in Western countries that emphasizes patient autonomy [44].

Doctors also expressed concerns about the beneficence of continuing not to speak directly with patients about prognosis and goals of care even as patients reached advanced stages of cancer. While doctors in our study described nondisclosure as intended by both themselves and families to protect patients, they also suspected it is not quite possible to hide the truth completely as advanced cancer symptoms become increasingly visible. This suspicion is supported by a 2021 survey of Chinese cancer patients showing that 19.7% inferred their diagnosis even when not directly told [45]. Chinese bioethicist Jing-Bao Nie suggests that seriously ill patients who secretly infer the truth likely experience more suffering than when informed directly—due to increased mistrust toward medical providers, barriers to open communication with loved ones, and isolation in coping silently with the possibility of dying [46]. Fear of causing patients to lose hope is a nearly universal barrier to truth-telling among doctors across cultures [47–49]. But there is evidence to suggest earlier palliative care discussions may increase patients’ hope in the long-run by improving the doctor-patient relationship, and redirecting patients and families toward more realistic goals [50, 51]. It is also important to notice how strongly doctors in our study emphasized the need to avoid conflict with families, in communication about palliative care. A top Chinese hospital, Peking Union Medical College Hospital, recently began piloting a palliative care consultation service that facilitates family meetings. Physicians using the service perceived decreases in patient-family anxiety, and relief of tension in the doctor-family-patient relationship [52]. Replicating family meetings as a palliative care service in other hospitals may help to diffuse doctor-family conflict, while also addressing ethical concerns about both patient autonomy and beneficent communication.

Another novel finding in this study was the difference in physicians’ perceptions of their own roles in discussions of goals of care. While several doctors in our study strove to advise objectively, others expressed awareness that, as experts, the way they presented options would influence the decisions of patients and families. Research from other countries supports the assumption of the latter group, showing physicians’ tone and level of comfort discussing palliative care can influence patients’ views of palliative care and the likelihood of initiating it, respectively [6, 53]. Evidence also suggests that higher awareness among physicians of their own mortality, spirituality, and emotions about death may help facilitate more open palliative care conversations and help physicians to be more present with dying patients [54–56]. Communication training for palliative care in China should address physicians’ self-awareness of their own tone and attitudes in palliative care conversations.

Recommendations have emerged in other countries such as the US encouraging doctors to take an active, guiding role in shared decision-making for palliative care [57]. To our knowledge, no comparable guidelines have been developed specifically for Chinese culture, and a 2015 systematic review concluded that research on shared decision-making and patients’ preferred roles in medical decisions is very limited in mainland China [58]. A 2018 survey on the preferences of Chinese cancer patients, family members, and physicians regarding various dimensions of breaking bad news showed that both patients and families ranked “information about the recommended treatment” as their fifth highest priority in receiving bad news, while doctors ranked the same item as the ninth highest priority [59]. This finding concurs with the hesitation of many doctors in our study to play an active, advisory role in discussions about goals of care. In order to develop clear, culturally competent guidelines in China, more research is needed to understand the preferences of patients and families regarding the role of the physician in discussions about goals of care for patients with advanced cancer and other serious illnesses.

Regardless of how influential they perceived their own role to be, almost all doctors in our study implied the family has the most authority in discussions about goals of care. Several doctors in our interviews believed public education about palliative and end-of-life care could help to resolve tension between doctors and families. Previous research has shown stigma associated with talking about death is a main barrier to palliative care use in China [13], and knowledge of palliative care among the Chinese public is generally low [2]. Studies in other countries have shown interventions such as informational pages or videos can improve knowledge of palliative care among laypersons [60]. Some hospitals and advocacy organizations in China have also conducted campaigns to publicize palliative care and encourage families to talk about death [13]. Future research should assess efficacy of such approaches, and how they may be further developed to improve palliative care awareness and communication in China.

### Limitations

This study should be interpreted in light of certain limitations. While participants were diverse in experience level, job status, and education level, the majority of oncologists and hematologists interviewed were female (87.5%). This was largely due to the hospital where the study was conducted having more female than male physicians in these specializations. Future studies may evaluate possible differences in perceptions of palliative care integration associated with gender. Furthermore, interviews were conducted at a tertiary hospital with no specialized palliative care unit, which may have limited generalizability to other care contexts in China.

## Conclusions

This study revealed conflicting views among physicians on the purpose and timing of palliative care for advanced cancer patients and the role doctors should play in discussions about goals of care. Doctors also experienced ethical tension between families’ preferences not to inform patients about advanced cancer and patients’ “right to decide” between treatment options. There is a need for training to standardize physicians’ understanding of palliative care and address ethical challenges in communication and discussions about goals of care. Medical training in China should encourage earlier integration of palliative care into treatment for advanced cancer. Communication skills training may also help equip doctors to mediate between preferences of patients and their families regarding information disclosure and the role of the physician. At the same time, wider use of family meetings and public education on palliative and end-of-life care may foster greater openness in palliative care communication.

## Data Availability

The data generated and analyzed during the current study are available from the corresponding author on reasonable request.
